# Fecal microbiota transplant as treatment for recurrent urinary tract infections: a proof-of-concept study

**DOI:** 10.1007/s10096-025-05202-9

**Published:** 2025-07-02

**Authors:** Verónica Rico-Caballero, Mario Romero-Rivera, Ana Moreno-Blanco, Andrea Aira, Climent Casals-Pascual, Concepción Rodríguez-Jiménez, Carmen Quereda, Alex Soriano, Rosa del Campo

**Affiliations:** 1 Servicio de Enfermedades Infecciosas, Hospital Clínic de Barcelona, Universidad de Barcelona, Barcelona, Spain; 2 Instituto de Investigación Biomédicas Agustí Pi Suñer, Barcelona, Spain; 3 Servicio de Microbiología, Hospital Universitario Ramón y Cajal, and IRYCIS, Madrid, Spain; 4 CIBER de Enfermedades Infecciosas, Instituto de Salud Carlos III, Madrid, Spain; 5 Servicio de Microbiología, Hospital Clinic de Barcelona, Universidad de Barcelona, Barcelona, Spain; 6 Servicio de Enfermedades Infecciosas, Hospital Universitario Ramón y Cajal, and IRYCIS, Madrid, Spain; 7 Facultad de Ciencias de la Salud, Universidad Alfonso X El Sabio, Villanueva de la Cañada, Madrid, Spain

**Keywords:** Urinary infection tract, Fecal microbiota transplantation, Gut reservoir of uropathogens

## Abstract

Recurrent urinary tract infections (rUTIs) are a global problem due to their high prevalence and because are caused by antibiotic-resistant strains. The hypothesis of our study is that a fecal microbiota transplant (FMT) might reduce the presence of uropathogenic *E. coli* in the intestinal microbiota, thereby leading to a decrease in the rUTIs. The objective of this study was to assess the efficacy of a single FMT intervention for the treatment rUTIs. A total of 22 postmenopausal women received a 3-day course of oral rifaximin to reduce their commensal microbiota, followed by a 2-day washout period, and then an oral FMT using lyophilized fecal capsules equivalent to 100 g of freeze-dried fecal matter from a single certificated fecal donor. The results demonstrated that over a period of six months, there was a 68.1% reduction in urinary infectious episodes, with minimal adverse effects. In addition, eradication of fecal colonization of urinary pathogens that were resistant to multiple antibiotics was achieved. The findings of this pilot study indicate that FMT is a promising and safe alternative for the treatment of rUTIs, particularly those caused by multidrug-resistant bacteria.

**Supplementary Information:**

The online version contains supplementary material available at 10.1007/s10096-025-05202-9.

## Introduction

Recurrent urinary tract infections (rUTIs) are a major public health challenge, with consequences that include strained healthcare resources, promotion and selection of multidrug-resistant (MDR) bacteria, and reduced quality of life. rUTIs are defined by the existence of 3 or more episodes in the past 12 months or 2 episodes in the last 6 months. This pathology exhibits a sex-biased distribution, with women constituting the demographic most affected, which can be attributed to anatomical and biological factors [10, 2].

A comprehensive understanding of the pathophysiology of rUTI is imperative for the development of effective eradication methods to prevent recurrrences. The gastrointestinal tract functions as a reservoir for uropathogens, which invade the vaginal mucosa and periurethral epithelium, ascending to colonize the bladder [4, 7, 11]. Furthermore, an independent risk factor for UTI in kidney transplant patients has been identified as the abundance of uropathogens in the gut [5]. Additionally, the vaginal microbiota has been demonstrated to limit bacterial exchange between the gut and the urinary tract by lactic acid production [7]. However, this protective effect is significantly diminished after menopause.

Urinary tract infections (UTIs) are primarily caused by Gram-negative bacteria, such as *Escherichia coli*,* Klebsiella* spp., *Proteus mirabilis*, and *Pseudomonas aeruginosa* [10]. There has been an alarming rise of antimicrobial resistance in these bacteria, including significant resistance to third-generation cephalosporins and carbapenems. The World Health Organization and the Centers for Disease Control and Prevention have classified uropathogens as critical microorganisms for which new antibiotics or treatment strategies are urgently needed. Current treatment strategies are primarily informed by a paucity of comprehensive guidelines and are largely predicated on expert opinion, frequently incorporating a combination of pharmacological interventions and other therapeutic modalities. Nevertheless, the efficacy of antibiotics in treating acute infections and preventing recurrence when administered in low doses on a chronic basis remains unparalleled.

Fecal microbiota transplant (FMT) has been demonstrated to be an effective treatment for recurrent *Clostridioides difficile* infection (rCDI). Initially, the microbiome from a healthy donor was transferred via colonoscopy or nasojejunal tube; however, current trends include lyophilization, with subsequent administration by oral capsules. Recent studies have demonstrated that FMT not only cures rCDI but can also reduce or even eliminate rUTIs [1, 9]. Our empirical observation in patients who had undergone FMT for rCDI, but who also had long-standing chronic rUTIs, suggests that the intervention provokes a ‘beneficial side-effect’ with clinical success in both pathologies. The objective of the present pilot study was to evaluate the potential role of FMT in the treatment of rUTIs and to assess the safety and the feasibility of the treatment in a proof-of-concept study.

## Results

A total of 22 postmenopausal women, with a mean age of 67 years, were recruited in both hospitals (see Table 1). It is noteworthy that 81.8% of the participants had previously undergone antibiotic prophylaxis for rUTIs. The patients had experienced an average of 5 rUTI episodes, ranging from 3 to 12, in the six months prior to FMT, with *E. coli* and *K. pneumoniae* being the most identified pathogens. Furthermore, multidrug-resistant uropathogens were identified in the urine of 9 (40.9%) women, including in 6 cases extended-spectrum beta-lactamase (ESBL)-producing Enterobacteriaceae. It is noteworthy that, except for three women, all patients had previously attempted various prophylactic strategies without success, including low-dose antibiotics for a period of six months.

**Table 1:** Patient characteristics and UTI episodes pre and post FMT

Patient number	Age	Comorbidities	Prophylaxis strategies previous FMT	UTI episodes previous/after FMT	Main rUTIs pathogen	ESBL eradication
1	77		Cranberries, mannose, vaccine, antibiotics	3 / 1	CP-*Pseudomonas aeruginosa*	No
2	63	cystocele	Cranberries, mannose, antibiotics	4 / 0	*Escherichia coli*	-
3	79	UI	Cranberries, mannose, vaccine, antibiotics	5 / 1	*Enterococcus faecalis*	-
4	74	UI	Cranberries, mannose, vaccine, antibiotics	5 / 1	*Enterobacter cloacae*	-
5	84	urolithiasis	Antibiotics, vaccine	4 / 1	*K. pneumoniae*	-
6	69	urolithiasis	Antibiotics	5 / 1	*E. coli*	-
7	71	UI, cystocele	Cranberries, antibiotics	12 / 1	ESBL-*Morganella morganii*	No
8	54	UI	Cranberries, mannose, antibiotics	3 / 3	ESBL-*K. pneumoniae*	Yes
9	85	UI	Antibiotics	5 / 2	ESBL-*K. pneumoniae*	No
10	50	UI, neurological bladder	Antibiotics	3 / 0	*E. coli*	-
11	72	UI	Antibiotics, vaccine	5 / 4	ESBL-*Escherichia coli*	No
12	77	UI, urolithiasis	No	6 / 4	AmpC-*Citrobacter koserii* and *E. coli*	-
13	73	UI, bladder diverticula	Antibiotics	3 / 1	ESBL-*K. pneumoniae*	Yes
14	59	cystocele	Antibiotics	8 / 1	*E. coli*	-
15	60		No	4 / 3	*E. coli* and *K. pneumoniae*	-
16	68	UI, emphysematous bladder	Antibiotics, vaccine	5 / 2	*E. coli*	-
17	83	UI, cystocele	No	4 / 3	ESBL-*K. pneumoniae*	Yes
18	53	urolithiasis	Antibiotics	6 / 0	AmpC-*E. coli*	-
19	60	Cystocele	Antibiotics	3 / 1	*Proteus mirabillis*	-
20	59	UI, cystocele	Antibiotics	3 / 2	*E. coli*	-
21	49		Antibiotics	7 / 2	*E. coli*	-
22	68	UI, urolithiasis	Cranberries	5 / 2	*E. coli*	-

UI: urinary incontinence

### FMT adverse effect and clinical follow-up

All participants reported good tolerance to FMT capsules, with no significant side effects. However, mild gastrointestinal adverse effects, including nausea, bloating, or abdominal pain, were reported by 40.9% of the participants, with all symptoms resolving within a few days. Constipation was reported in 3 patients during the 6-month follow-up period. Following the intervention, a mean reduction in the number of rUTI episodes was observed, from a mean of 5 (range: 3–12) to 1.6 (range: 0–4). The primary endpoint, defined as a 50% reduction in episodes, was achieved by 68.1% of patients. The mean time to the next episode was found to be 26.6 days, and 3 patients had no further episodes (see Table 1 for details).

### Gut decolonization of antibiotic-resistant bacteria

Multidrug-resistant uropathogens detected at basal time included ESBL-producing *K. pneumoniae* (*n* = 4), *E. coli* (*n* = 1), and *Morganella morganii* (*n* = 1), as well as carbapenemase-producing *Pseudomonas aeruginosa* (*n* = 1). After the FMT intervention, UTIs recurrences were caused by antibiotic-susceptible bacteria, and gut decolonization of ESBL-producing *K. pneumoniae* was corroborated in 3 out of the 4 cases (Table 1).

### Gut microbiota characterization

**Fig. 1:**
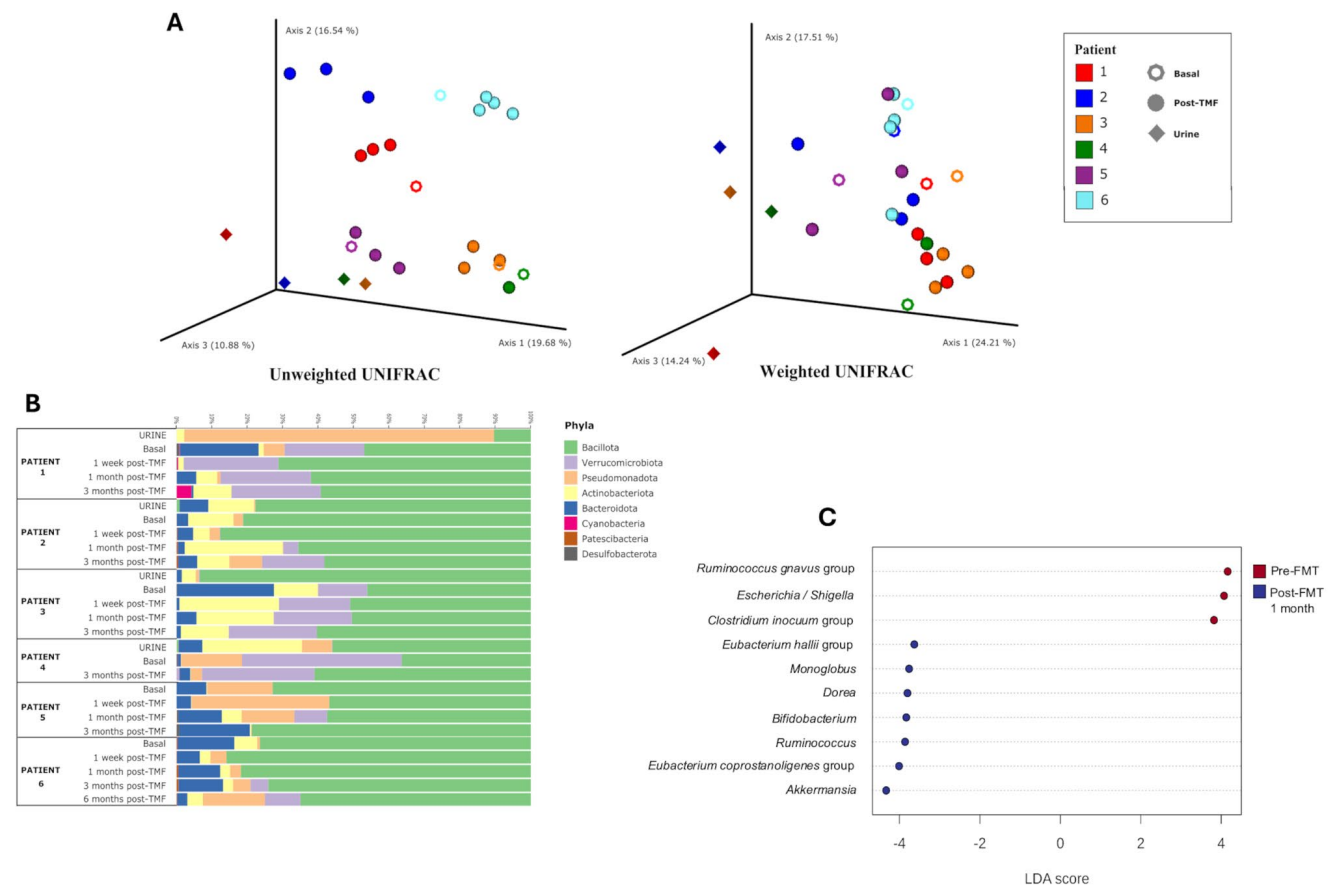
Composition of the microbiome before and after FMT. **A**: weighted and unweighted UNIFRAC showing clustering distances between samples for each patient and in relation to TFM. **B**: variation in phylum abundance for each sample. **C**: LEfSe analysis highlighting the main changes in genus abundance for all patients before and after TFM

The clinical outcome of the 6 women whose microbiota composition was characterized was satisfactory in 4 cases, while the remaining 2 did not meet the target of halving the number of recurrences. Previous infections were caused by *E. coli* (3 successful and 1 unsuccessful), *P. mirabilis* (1 successful) and ESBL-producing *K. pneumoniae* (recurrence of UTI but ESBL eradication). No significant differences were observed in the changes in the microbiota of the 6 women, with each showing particular changes after FMT administration (Fig. 1). The overall changes were a fourfold increase in *Akkermansia* (phylum Verrucomicrobia) and a fourfold decrease in *E. coli* and phylum Pseudomonadota. Opposite trends were observed in the *Ruminococcus* genera; while the overall population increased significantly, the density of *Ruminococcus gnavus* species decreased (Fig. 1).

## Discussion

The present study corroborates previous research suggesting that FMT is a promising alternative to antibiotics for the treatment of rUTI. A 50% reduction in recurrences was observed in 68.1% of treated patients during the six months following FMT. These outcomes are consistent with those reported in the most extensive study to date by Tariq et al., [9] which found that eight patients with rCDI and rUTI experienced a 50% reduction in UTI episodes following FMT. A recent study [3] investigated the restoration of the vaginal and gut microbiota using probiotics, with significantly reduction in the number of rUTI. In patients with rCDI, the engraftment of the new microbiota following FMT is effective because there is an empty ecological niche with few competitors for nutrients, which explains the rapid restoration of the microbiota and the positive outcome associated with FMT in rCDI [6]. Conversely, patients with rUTI exhibit a robust microbiota, characterized by a substantial representation of the *phylum* Pseudomonadota. To mitigate this, a course of rifaximin was administered prior to FMT, and both interventions resulted in a significant reduction of the Pseudomonadota *phylum* to approximately a quarter of its original levels. Moreover, the eradication of antibiotic-resistant isolates signifies that certain clones are being replaced by others. Conversely, studies have found that antibiotic prophylaxis may increase the risk of new UTIs caused by MDR pathogens, making FMT a preferable alternative [8]. The observed changes may provide a rationale for the emergence of FMT as a promising therapeutic strategy, in accordance with the current understanding of the pathophysiology of UTI, in which the composition of the microbiota plays a pivotal role [5, 7].

Notwithstanding the encouraging results, there are certain limitations to the present study. The absence of a control group, the limited sample size, and the restricted analysis of the microbiota in non-responders all make it difficult to draw definitive conclusions. It is recommended that a subsequent study be conducted in the future, with consideration given to the variability of FMT donors and recipients. Furthermore, the optimization of the FMT protocol and dose, as well as the assessment of the long-term effects, should be prioritized.

## Methods

### Study population and specimen collection

Women aged 18 or older with a history of rUTIs were recruited at the Infectious Diseases Departments of the University Hospitals Clínic (Barcelona) and Ramón y Cajal (Madrid), Spain. Participants were monitored for 6 months; urine and stool samples were collected at baseline, and at 1 week, 1 month, 3 months, and 6 months post-FMT. In addition, feces and urine samples were collected when the patient reported a possible UTI. After collection, samples were immediately aliquoted and frozen at -80ºC until processing. Patients were followed up by direct telephone contact, and clinical examinations and questioning were performed each time the patient came to the hospital.

### Inclusion/exclusion criteria

The women were eligible if they had antibiotic allergies, infections with limited or no oral treatment options (ESBL- or carbapenemase-producing Enterobacteriaceae, and quinolone-resistant *Pseudomonas aeruginosa* or *Enterococcus*), or failure after prolonged antibiotic prophylaxis. Exclusion criteria included active UTI symptomatology, ongoing antibiotic prophylaxis, pregnancy, breastfeeding, immunocompromised conditions, uncontrolled illnesses, or inflammatory bowel disease.

### Ethics statement

The study protocol was approved by the ethics committee of the Hospital Ramón y Cajal under reference 137/19, with subsequent validation by the ethics committee of the Hospital Clínic. Written informed consent was obtained from all participants.

### Recurrence UTI episode and outcome definitions

The criterion for considering a recurrence of UTI was the coexistence of clinical symptomatology with a positive quantitative urine culture. The primary outcome was to achieve at least a halving of the number of episodes in the 6 months of monitoring compared to the 6 months prior to the intervention. The secondary outcome was to monitor the modification of the gut microbiota and the decolonization of the uropathogens and/or the genetic mechanism codifying the antibiotic resistance.

### FMT procedure

The participants received a 3-day course of oral rifaximin (1,200 mg/day) to reduce and deconstruct their native gut microbiota. This was followed by a 2-day washout period, after which a single dose of 100 g of lyophilized fecal material was administered via oral FMT. To standardize results, a single volunteer was carefully examined and confirmed to be a fecal donor, paying particular attention to the absence of antibiotic-resistant microorganisms in their feces by culture with selective media.

### Microbiome analysis and statistical analysis

Urine samples were routinely cultured to detect the usual pathogens. The composition of the fecal microbiota was determined in a subset of six patients from the same center, who were the only subjects in whom samples had been collected for this purpose. Fecal and urine aliquots were gradually defrosted at a temperature of − 20 °C for 24 h, followed by a further 24 h at 4 °C. DNA were extracted using a QiaAMP kit (Qiagen, Germany). 16 S rDNA sequencing was conducted following the selective amplification of the variable regions V3 and V4 of the 16 S rRNA using the Illumina Miseq platform (Illumina Inc., USA) with a read length of 2 × 300 bp. The sequences with low quality, quimeric or small size were filtered using the DADA2 algorithm and the resulting features were assigned as representative amplicon sequence variants (ASVs). Classification was performed using the SILVA 138 sequence classifier. The microbial composition was studied using the MicrobiomeAnalyst web server with default parameters, alpha diversity was estimated using the Shannon and Chao1 indexes, and beta diversity by Bray-Curtis index. In addition, clinical data were analyzed using the Statistical Package for Social Sciences (SPSS), with continuous variables reported as medians, and categorical variables as percentages.

## Electronic supplementary material

Below is the link to the electronic supplementary material.

Supplementary Material 1

Supplementary Material 2

## Data Availability

16 S rDNA sequences are deposited under the BioProject ID: PRJNA1222286 accesion.
