# Supplementary material for: Fecal microbiota transplant as treatment for recurrent urinary tract infections: a proof-of-concept study
Source: Eur J Clin Microbiol Infect Dis. 2025 Jul 2;44(10):2549–54. doi: 10.1007/s10096-025-05202-9 (PMC12484344; doi:10.1007/s10096-025-05202-9)
Supplement: Supplementary file 1 — Supplementary Material 1 [file 10096_2025_5202_MOESM1_ESM.docx]

**Table 1.** Patient characteristics and UTI episodes pre and post FMT.

| Patient number | Age | Comorbidities | Prophylaxis strategies  previous FMT | UTI episodes  previous/after FMT | Main rUTIs pathogen | ESBL eradication |
| --- | --- | --- | --- | --- | --- | --- |
| 1 | 77 |  | Cranberries, mannose, vaccine, antibiotics | 3 / 1 | CP-*Pseudomonas aeruginosa* | No |
| 2 | 63 | cystocele | Cranberries, mannose, antibiotics | 4 / 0 | *Escherichia coli* | - |
| 3 | 79 | UI | Cranberries, mannose, vaccine, antibiotics | 5 / 1 | *Enterococcus faecalis* | - |
| 4 | 74 | UI | Cranberries, mannose, vaccine, antibiotics | 5 / 1 | *Enterobacter cloacae* | - |
| 5 | 84 | urolithiasis | Antibiotics, vaccine | 4 / 1 | *K. pneumoniae* | - |
| 6 | 69 | urolithiasis | Antibiotics | 5 / 1 | *E. coli* | - |
| 7 | 71 | UI, cystocele | Cranberries, antibiotics | 12 / 1 | ESBL-*Morganella morganii* | No |
| 8 | 54 | UI | Cranberries, mannose, antibiotics | 3 / 3 | ESBL-*K. pneumoniae* | Yes |
| 9 | 85 | UI | Antibiotics | 5 / 2 | ESBL-*K. pneumoniae* | No |
| 10 | 50 | UI, neurological bladder | Antibiotics | 3 / 0 | *E. coli* | - |
| 11 | 72 | UI | Antibiotics, vaccine | 5 / 4 | ESBL-*Escherichia coli* | No |
| 12 | 77 | UI, urolithiasis | No | 6 / 4 | AmpC-*Citrobacter koserii* and *E. coli* | - |
| 13 | 73 | UI, bladder diverticula | Antibiotics | 3 / 1 | ESBL-*K. pneumoniae* | Yes |
| 14 | 59 | cystocele | Antibiotics | 8 / 1 | *E. coli* | - |
| 15 | 60 |  | No | 4 / 3 | *E. coli* and *K. pneumoniae* | - |
| 16 | 68 | UI, emphysematous bladder | Antibiotics, vaccine | 5 / 2 | *E. coli* | - |
| 17 | 83 | UI, cystocele | No | 4 / 3 | ESBL-*K. pneumoniae* | Yes |
| 18 | 53 | urolithiasis | Antibiotics | 6 / 0 | AmpC-*E. coli* | - |
| 19 | 60 | Cystocele | Antibiotics | 3 / 1 | *Proteus mirabillis* | - |
| 20 | 59 | UI, cystocele | Antibiotics | 3 / 2 | *E. coli* | - |
| 21 | 49 |  | Antibiotics | 7 / 2 | *E. coli* | - |
| 22 | 68 | UI, urolithiasis | Cranberries | 5 / 2 | *E. coli* | - |

UI: urinary incontinence.
