# Supplementary figures and images for: Fecal microbiota transplant as treatment for recurrent urinary tract infections: a proof-of-concept study
Source: Eur J Clin Microbiol Infect Dis. 2025 Jul 2;44(10):2549–54. doi: 10.1007/s10096-025-05202-9 (PMC12484344; doi:10.1007/s10096-025-05202-9)

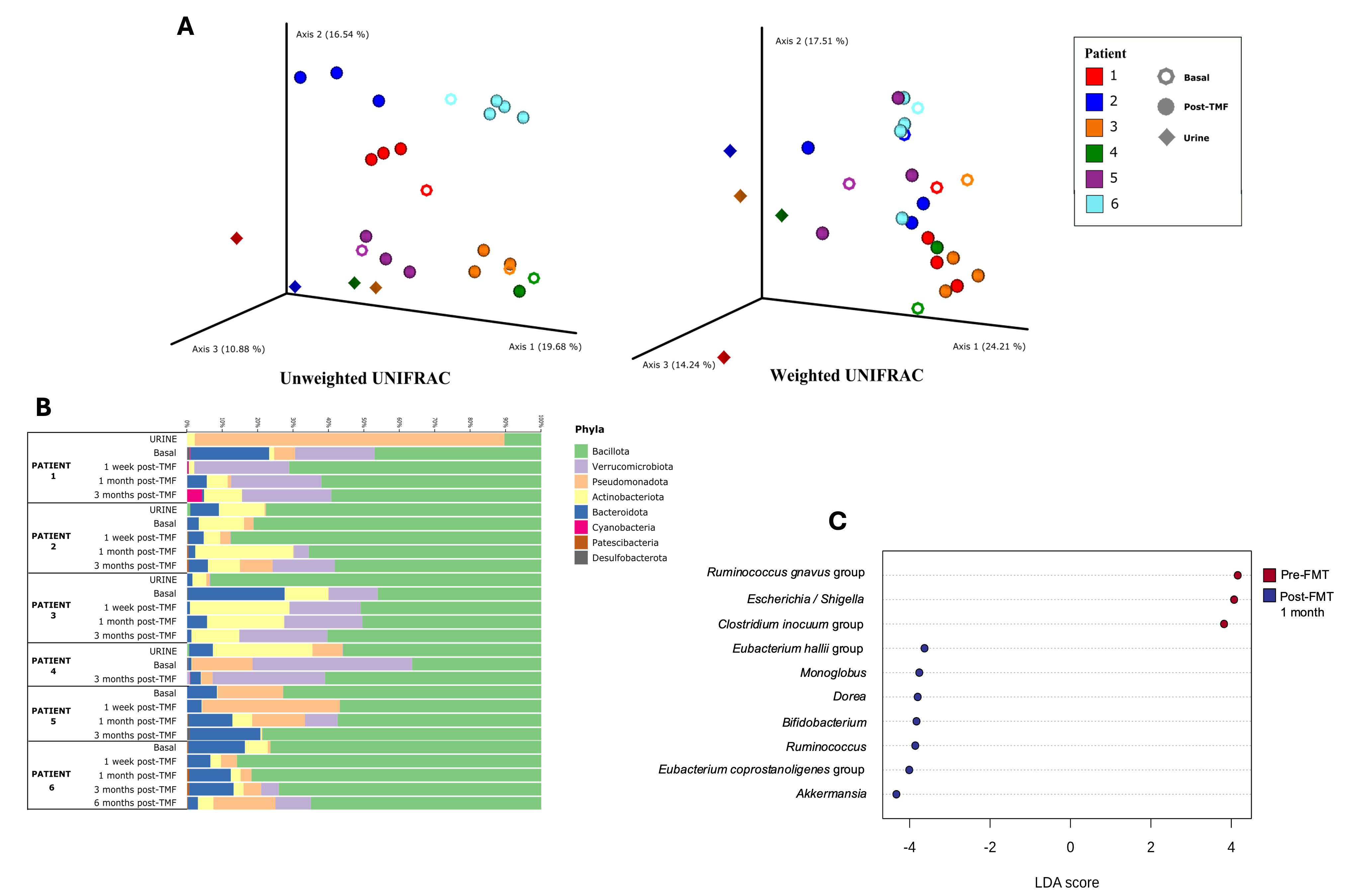

Supplement: Supplementary file 2 — Supplementary Material 2 [file 10096_2025_5202_MOESM2_ESM.png]
